# The Effect of EMG Features on the Classification of Swallowing Events and the Estimation of Fluid Intake Volume

**DOI:** 10.3390/s22093380

**Published:** 2022-04-28

**Authors:** Carlotta Malvuccio, Ernest N. Kamavuako

**Affiliations:** 1 Department of Engineering, King’s College London, London WC2R 2LS, UK; carlotta.1.malvuccio@kcl.ac.uk; 2 Faculté de Médecine, Université de Kindu, Site de Lwama II, Kindu, Maniema, Congo

**Keywords:** surface electromyography, swallowing events, geriatrics, hydration, fluid intake

## Abstract

Nowadays, society is experiencing an increase in the number of adults aged 65 and over, and it is projected that the older adult population will triple in the coming decades. As older adults are prone to becoming dehydrated, which can significantly impact healthcare costs and staff, it is necessary to advance healthcare technologies to cater to such needs. However, there has not been an extensive research effort to implement a device that can autonomously track fluid intake. In particular, the ability of surface electromyographic sensors (sEMG) to monitor fluid intake has not been investigated in depth. Our previous study demonstrated a reasonable classification and estimation ability of sEMG using four features. This study aimed to examine if classification and estimation could be potentiated by combining an optimal subset of features from a library of forty-six time and frequency-domain features extracted from the data recorded using eleven subjects. Results demonstrated a classification accuracy of 95.94 ± 2.76% and an f-score of 94.93 ± 3.51% in differentiating between liquid swallows from non-liquid swallowing events using five features only, and a volume estimation RMSE of 2.80 ± 1.22 mL per sip and an average estimation error of 15.43 ± 8.64% using two features only. These results are encouraging and prove that sEMG could be a potential candidate for monitoring fluid intake.

## 1. Introduction

The indisputable fact that our society faces nowadays is the increase in the population aged 65 and over. According to the United Nations [1], by 2050, one in six people will be aged over 65. The number of persons aged 80 years and over is projected to triple. This increase in the number of older adults is primarily due to societal improvements in lifestyle and advances in technology and healthcare. Nevertheless, it will soon pose new challenges if action is not taken to advance the current monitoring technology, as older adults necessitate more care and medications. One challenge in our healthcare system is dehydration, which is a recurrent issue in older adults [2,3]. Dehydration is mainly provoked by a diminished thirst sensation with ageing and mobility or memory impairments [4,5]. Moreover, light hydration in older adults increases the chances of falls and hence the possibility of bone fractures, other than potentially leading to drug intoxication, confusion, and delirium in severe cases [6,7,8]. Furthermore, it has been recently suggested that chronic suboptimal hydration might be a risk factor for increased mortality in COVID-19 patients on top of risk factors such as male gender and older age [9].

There are no gold standards for identifying dehydration [10,11]. Moreover, signs of dehydration, such as skin turgor, are often confounded by the age of the patients, and assessing dehydration is made difficult by a shortage of staff, absence of appropriate laboratory analyses and memory impairment of senile patients [12,13]. Thus, the best option is to ensure that older adults intake the recommended daily fluid volume [14,15]. To keep track of service users’ fluid intake, nurses must fill manually fluid monitoring charts, contributing to a substantial increase in their workload. This method is inaccurate and prone to misreporting, as nurses mainly rely on patient information and relatives rather than direct observation of the patient [16]. Thus, maintaining adequate oral hydration for older residents is an ongoing challenge for staff members, bringing the prevalence of dehydration in nursing homes residents at 37% in the United Kingdom (UK) alone [17], adding strain to healthcare costs. For example, Xiao et al. [18] reported that the average length of stay for dehydration in the Healthcare Cost and Utilisation Project (HCUP) data was 4.6 days, amounting to a total hospitalisation charge per person of $7442. Therefore, there is an urgent need to develop accurate methods to monitor fluid intake in older adults to ease the burden on healthcare staff and healthcare finances. 

Only a handful of studies in the literature have tried to develop fluid intake monitoring systems using signals harvested directly from the subjects. Most of these used microphones only [19,20], and data collection was performed on a very limited number of participants. Amft and Tröster [21] combined surface electromyography (sEMG) with microphones to perform classification between solid and liquid foods. The reported classification accuracy was less than 84%. Apart from this paper, to the best of our knowledge, there is no other record in the literature of sEMG used with the specific scope of quantifying fluid intake. Indeed, most of the documentation found in the literature refers to sEMG being used to study dysphagia, although some of these studies hinted at the possibility that certain neck sEMG features such as peak amplitudes change according to the swallowed volume [22,23]. Therefore, we conducted a preliminary study on swallowing events classification and volume estimation using four predefined time-domain features. The aim was to assess if neck sEMG could distinguish between saliva and liquid swallows and then if it was possible to perform volume estimation. The results of our published study [24] showed a mean classification accuracy between saliva and liquid swallows of 86.69 ± 5.52% using the k-Nearest Neighbour classifier. The average Root Mean Square Error was 2.01 ± 1.39 mL per swallowed sip using an Artificial Neural Network (ANN) with two hidden layers, each with fifteen neurons. 

While these results were encouraging, it was necessary to investigate further whether classification and volume estimation could be improved using a more comprehensive set of features and studying what could be the ideal combination to obtain the best performance possible. Again, the need to carry out this investigation stemmed from the fact that there is no record in the literature about an extensive analysis of neck sEMG features. Hence, we extracted forty-six single features, of which thirteen were frequency-domain, using the list of features detailed in the paper from Phyniomark et al. [25], and we applied these on the sEMG of neck muscles involved during the swallowing events. 

## 2. Materials and Methods

The Research Ethics Committee of King’s College London (LRS-18/19-10877) approved this study. We recruited eleven healthy participants (3 F, 8 M, age range from 20 to 67 years) with no known underlying medical conditions. All participants agreed voluntarily to participate in the experiment, and written informed consent was obtained.

**Figure 1:**
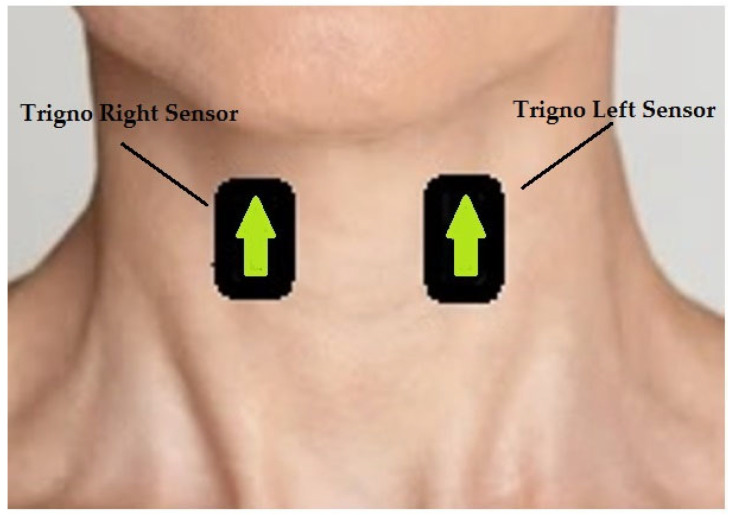
The figure shows the anatomical position on which sensors were placed. The arrows indicate the use of Delsys trigno system.

Participants sat comfortably, and the skin around the neck was prepared using alcohol wipes. Two Delsys Trigno sensors (Delsys Incorporated, Natick, MA, USA) were set as sEMG and placed on the belly of the sternohyoid muscles (infrahyoid group), as shown in Figure 1. The sternohyoid muscles were chosen, as these are the most superficial muscles of the infrahyoid group. The correct anatomical placement of the sensors was identified via palpation of the neck muscles of the participants, as the distancing and position were highly dependent on the sex and body conformation of participants. We collected data collection using two pieces of equipment. A TREE KHR502 electronic scale (resolution of 0.01 g and a capacity of 500 g) was used to measure the volume of water ingested by the subjects during each task. The Delsys Trigno wireless EMG system (gain: 42 V; bandwidth: 20–450 Hz, sampling rate: 1 kHz) collected the sEMG signals produced during the swallowing process. The length of each recording was manually set as 10 s.

Once sensors were placed on the correct anatomical positions, subjects were asked to perform seven tasks, with six including water at room temperature. The subject was instructed to swallow for each task following a cue. The time between cues was at the participant’s discretion, and the recording was initiated two seconds prior to each cue. The first task consisted of performing one saliva swallow, which was repeated five times. Tasks II, III, and IV consisted of ingesting water from the administered container five times. For each of these three tasks, the container used by the subject was randomly changed in a cup (Task II), bottle (Task III) and straw (Task IV), respectively, and subjects were instructed to sip as they would normally do in real-life scenarios. The volume of each sip was calculated using the laboratory scale. The selected fluid container was filled and placed on the scale, and we noted the initial weight of the container. We cued each subject to take a single sip as typically as possible and place the container back on the scale. We then subtracted the final weight from the initial weight to note the swallowed volume. Thus, we did not impose specific volumes, and the only condition was to take a single sip. Task V consisted of the participant being administered a total liquid volume corresponding to the highest volume ingested in Task II plus 5 mL and was performed once. This task had the scope of improving linear regression and volume estimation. Adding 5 mL to the highest ingested volume would ideally produce an observation corresponding to the maximum swallowing capacity of the subject.

**Figure 2:**
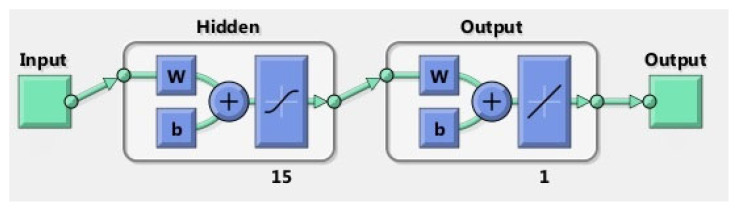
The figure shows the diagram of the ANN model used in this study.

The collected EMG signals were processed using the MATLAB R2020b version. First, a Kaiser window FIR bandpass filter with a bandpass frequency range of 20 to 400 Hz, transition band steepness of 0.85, and stopband attenuation of 60 dB was applied to the left and right sternohyoid signals. In order to perform burst extraction and identify the burst region, the signal was smoothed using a moving RMS envelope with a window length of 1000 ms. These two steps are illustrated by the graphs shown in Figure 2. The burst region was identified by locating the peak with the highest value of the smoothed signal and including the 750 data points (0.75 s) to the left and right of the highest peak, thus resulting in a total burst duration of 1500 ms. Once the burst location was identified, the burst was extracted from the raw bandpass filtered signal. Finally, baseline noise was extracted by taking the last 1.5 s of the recorded signal that contained no swallowing information based on throughout visual inspection of the signals. A total of forty-six single features, thirty-three time-domain and thirteen frequency-domain features were computed from each 1500 ms window (burst and the baseline noise). The features used were selected based on the paper from Phinyomark et al. [25] and presented in Table 1.

Features were calculated per 1500 ms window to represent a single sip. As this study aimed to verify which features combination performs best for our scope, stepwise forward selection was used. Each problem (classification or estimation) was performed using single features. The feature with the highest performance metric was retained as the best feature and then combined with each of the remaining 45 features and tested in pairs. The pair with the highest performance metric was then combined with each of the remaining 44 features, and then, the procedure was repeated until the performance parameters reached a plateau or did not improve further. A one-way analysis of variance (ANOVA) test was used to test if the resulting metrics significantly differed with an increasing number of features. 

The classification was modelled per subject as a two-class problem, with one class containing the baseline noise and saliva swallows’ data together (including 11 noise observations and five saliva bursts) against the class containing liquid swallows (a total of 16 observations). The classifiers employed were Linear Discriminant Analysis (LDA) and k-Nearest Neighbour with k = 1 (KNN). To estimate the performance of the classifiers, the Leave-One-Out Cross-Validation (LOOCV) method was used with the following performance metrics: Accuracy, Sensitivity, Specificity, Precision, and F-score, as presented in Equations (1)–(5). Accuracy quantifies the ability of the model to assign the observation to the correct class. Sensitivity measures the ability of the model to predict actual liquid swallows. Specificity measures the power of the model to correctly predict non-liquid swallowing events. Precision aims to quantify the proportion of liquid swallows classified as liquid swallows. Then, the F-score was calculated to assess if the model trade-off between precision and sensitivity was acceptable.

(1) $$\mathrm{Accuracy}=\frac{\mathrm{true}\;\mathrm{liquid}\;\mathrm{swallows}+\mathrm{true}\;\mathrm{non}\;\mathrm{liquid}\;\mathrm{events}}{\mathrm{number}\;\mathrm{of}\;\mathrm{obs}}$$

(2) $$\mathrm{Sensitivity}=\frac{\mathrm{true}\;\mathrm{liquid}\;\mathrm{swallows}}{\mathrm{liquid}\;\mathrm{swallows}}$$

(3) $$\mathrm{Specificity}=\frac{\mathrm{true}\;\mathrm{non}\;\mathrm{liquid}\;\mathrm{events}}{\mathrm{non}\;\mathrm{liquid}\;\mathrm{events}}$$

(4) $$\mathrm{Precision}=\frac{\mathrm{true}\;\mathrm{liquid}\;\mathrm{swallows}}{\mathrm{true}\;\mathrm{liquid}\;\mathrm{swallows}+\mathrm{false}\;\mathrm{liquid}\;\mathrm{swallows}}$$

(5) $$\mathrm{F-}\mathrm{Score}=\frac{2(\mathrm{precision}\;\times \;\mathrm{sensitivity})}{\mathrm{precision}+\mathrm{sensitivity}}$$

Before proceeding with more sophisticated methods to quantify fluid intake, first, we wanted to verify if the mean sip volume for each subject could be used to predict fluid intake within each subject. The mean and standard deviation of the sips taken by each subject were calculated, and the error was computed as the difference between the mean value as the predicted intake and actual sip volumes. 

Secondly, Linear Regression (LR) and a shallow Artificial Neural Network (ANN) were used to perform volume estimation analysis. The performance parameters used for both methods were Root Mean Square Error (RMSE) and mean estimation error as a volume percentage calculated using Equation (6):

(6) $$\mathrm{mean}\;\mathrm{EE}\;(\%)=\mathrm{mean}\left(\left|\frac{{\mathrm{predicted}}_{i}\;{-\;\mathrm{actual}}_{i}}{{\mathrm{actual}}_{i}}\right|\times \;100\right)$$

The linear regression model proposed in this study used the recorded sip volumes as response variables and the extracted features as predictor variables. The model specification was set to linear, meaning that the model contained an intercept and a linear term for each predictor, and it used ordinary least squares as a fitting method. Cross-validation was set to 5 k-fold. The ANN model consisted of one layer of 15 neurons with a hyperbolic tangent sigmoid transfer function and a linear transfer function for the output layer, as shown in Figure 2. Data division was performed at random, and the Levenberg–Marquardt algorithm was used for training. As previously described, the optimum feature subset was selected using the forward stepwise selection method. 

## 3. Results

### 3.1. Classification

The optimal classification performance was given by the KNN classifier using five features, namely: Integrated EMG (IEMG), Sign Slope Change (SSC), Average Amplitude Change (AAC), Area Under the Curve (AUC) and Variance of Central Frequency (VCF). However, results showed that the parameters of LDA classifier, when using five features, were declining compared to the use of four features (IEMG, SSC, AAC and AUC). These results are illustrated in Table 2. ANOVA tests demonstrated that the difference between four-feature and five-feature classification was statistically not significant both for LDA (*p* = 1) and KNN (*p* = 0.44). Furthermore, the ANOVA test demonstrated that while for LDA, there is only a significant difference between single-feature and four-feature classifier (*p* = 0.047), this changes for KNN. Indeed, the results showed that classifiers using one and two features are significantly different from those using three or more features (*p* < 0.05). Classifiers using three, four, and five features instead were not statistically different from each other (*p* > 0.05). 

### 3.2. Volume Estimation

The method of using the mean sip as the estimation volume resulted in large estimation errors, as shown in Table 3. When using LR for volume estimation, the best performance was given by a single feature, namely the Absolute value of the Summation of the exp^th^ root of the given signal and its Mean (ASM), with an RMSE of 3.90 ± 1.58 mL and an average estimation error of 24.63 ± 7.03% of the actual swallowed sip volume, as shown in Table 4. Concerning the ANN, the optimal volume estimation performance was given by SSC and Mean Power Density (MPD) combined with an RMSE of 2.80 ± 1.22 mL and an average estimation error of 15.43 ± 8.64% of the actual swallowed volume. Results for the ANN are illustrated in Table 5.

## 4. Discussion

The classification performances obtained in this study seemingly hint at the suitability of using surface EMGs to classify between liquid swallows versus non-liquid swallowing events. By analysing and determining an optimal set of features, classifier performance seemed to improve compared to the results obtained in our previous study [24] when a set of predefined features was used. Furthermore, in our previous study, signals coming from both the digastric and sternohyoid muscles were used, whereas in this study, only the sternohyoid signals were used. The fact that the performance has not deteriorated if classification is performed using data harvested from the left and right sternohyoid muscles alone hints that there is not a necessity to include digastric muscles in future studies. As the latter are submental muscles, the inclusion of surface EMGs in that anatomical region might create discomfort, even if minor, in the movements performed by subjects and even more so in hospitalised patients. Furthermore, digastric muscles are more sensitive to other movements, such as chewing and the swallowing preparation phase. These muscles are recruited to facilitate the ingress of food and fluids in the mouth, thus complicating the extraction of bursts related to fluid swallowing events. In terms of the optimal number of features, as summarised in Table 1, a KNN classifier using five features produces the best performance with an F-score of 94.93. However, statistical analysis demonstrated no significant difference between the performance of four features compared to five features. Therefore, while five features provided the highest accuracy, perhaps the choice of using four features could improve the computational time and cost, especially in continuous online tasks—an assumption that should be validated by potential future studies using larger cohorts. 

However, while these results seem promising and demonstrated an improvement on classification performance compared to previous studies [21,24], and they hint at the potential of surface EMGs to differentiate between liquid versus non-liquid swallows, these results need to be validated further. Further validation of these results could be performed by recruiting a larger number of subjects spanning a wide age range (also to verify if age has an influence on performance), as the results presented in this research are generated by a small number of participants; thus, it is necessary to observe if the performance will remain unchanged using a larger pool of participants; by harvesting a larger number of observations compared to the numbers collected in this study; and there is also the need to include in the non-liquid swallowing class more tasks such as coughing, talking, chewing and swallowing food of different viscosities to observe with significant certainty if surface EMGs possess the ability to distinguish all non-liquid swallowing events from liquid swallowing ones. 

The evidence gathered in this study and our previous one suggests that surface EMGs also have the potential to estimate fluid intake, thus being an alternative to the use of microphones for fluid estimation purposes [19,20]. Furthermore, if this potential will be corroborated in future studies, this will also confirm the observations of previous studies about the possibility of certain features derived from the neck surface EMGs to differentiate between swallows of different volumes [22,23]. The first method employed in this paper, which used the mean sip volume as an estimator, did not return satisfying results. The estimation error that resulted was larger than that of more sophisticated estimation methods such as using an ANN. This is mainly due to the fact that, as exposed in our previous study, the sip volume is influenced by the shape of the container when this is significantly different in size [24]. Indeed, sips ingested using the straw present a smaller volume compared to the ones consumed from bigger containers such as cups and bottles. Furthermore, factors such as the temperature of the liquid and composition (as an example, carbonated drinks), which were not considered in this study, could influence the sip volume, which might vary from subject to subject, rendering this method quite susceptible to estimation errors. Thus, these factors (fluid temperature and fluid composition such as carbonated drinks), which in real-life scenarios can be commonplace, would render this estimation method ineffective and inaccurate.

Using Linear Regression and ANN improved performance compared with the mean sip estimator with an RMSE of 3.90 ± 1.58 mL and an average estimation error of 24.63 ± 7.03% of the actual swallowed sip volume for the LR. ANN had an RMSE of 2.80 ± 1.22 mL and an average estimation error of 15.43 ± 8.64% for the ANN. As the two means are statistically different (*p* < 0.05), it could be concluded that the best volume estimation performance is given by the ANN. Furthermore, it is worth noting that the ANN developed in this study resulted in a similar performance compared to the two-layer, feed-forward network proposed in our previous study (average RMSE of 2.01 mL), which also used four features instead of two. Hence, this demonstrates that performance can be improved by selecting the appropriate features to combine while also using a network with a lower number of hidden layers, thus reducing the computational costs. We recommend further research to validate the hypothesis presented in this paper with a larger cohort and a more significant number of observations.

However, it is fundamental to reiterate that these findings need to be validated in future studies using a larger cohort that should span across age groups. Indeed, due to the restricted number of participants and limited age range, other factors such as the influence of age on mean sip volume could not be observed. Furthermore, while the classification results shown in this study were promising, there is a need to verify if the performance will remain optimal when including different liquid viscosities and other non-liquid events, such as talking and the ingestion of solid food.

## 5. Conclusions

The results obtained in this study hint at the potential of surface EMGs not only to differentiate between liquid and non-liquid swallows but also to estimate fluid intake using an optimum set of features. While further research is needed to cater for the limitations presented in this study, our findings could represent a way forward to produce a non-invasive device that could prevent dehydration in older adults and improve the quality of care in healthcare settings. 

## Figures and Tables

**Table 1:** This table presents the forty-six single features included in this study. The features are presented in the order that these were computed.

Feature Full Name	Abbreviation	Parameters
Integrated EMG	IEMG	-
Mean Absolute Value	MAV	-
Mean Absolute Value 1	MAV 1	-
Mean Absolute Value 2	MAV 2	-
Simple Squared Integral	SSI	-
Variance of EMG	VAR	-
Root Mean Square	RMS	-
Second V-Order	V2	v = 2
Third V-Order	V3	v = 3
Log Detector	LOG	-
Waveform Length	WL	-
Average Amplitude Change	AAC	-
Difference Absolute Standard Deviation Value	DASDV	-
Maximum Fractal Length	MFL	-
Myopulse Percentage Rate	MYOP	threshold = 5.5 μ
Willinson Amplitude	WAMP	threshold = 0.3 × σ (noise)
Modified Mean Absolute Value	MMAV	-
Zero Crossing	ZC	threshold = 0.3 × σ (noise)
Slope Sign Change	SSC	-
Abs. val. of Third Temporal Moment	TM3	order = 3
Abs. val. of Fourth Temporal Moment	TM4	order = 4
Abs. val. of Fifth Temporal Moment	TM5	order = 5
Abs value of the Summation of Square Root	ASS	-
Mean Value of Square Root	MSR	-
Absolute value of the Summation of the exp^th^ root of the given signal and its Mean	ASM	-
Kurtosis	Kurt	-
Skewness	Skew	-
Amplitude of the First burst	AFB	-
Mean Power	MNP	-
Total Power	TTP	-
Median Frequency	MDF	-
Mean Frequency	MNF	-
Peak Frequency	PKF	-
First Spectral Moment	SM1	order = 1
Second Spectral Moment	SM2	order = 2
Third Spectral Moment	SM3	order = 3
Frequency Ratio	FR	lc < MNF; hc > MNF
Mean Power Density	MPD	-
Power Spectrum Deformation	PSDd	-
Variance of Central Frequency	VCF	-
Higuchi Fractal Dimension	HFD	k = 128
Sample Entropy	SaEn	m = 2, r = 0.2 σ
Approximate Entropy	ApEn	m = 2, r = 0.2 σ
Maximum to Minimum Drop in Power Density Ratio	dPDR	-
Power Spectrum Ratio	PSR	*n* = 20
Area Under the Curve	AUC	-

**Table 2:** The table illustrates the resulting performance parameters for four features (IEMG, SSC, AAC and AUC) in the first row and five features (IEMG, SSC, AAC, AUC and VCF) in the second row.

LDA					KNN				
Accuracy	Sensitivity	Specificity	Precision	F Score	Accuracy	Sensitivity	Specificity	Precision	F Score
95.80 ± 4.62	96.02 ± 6.42	95.61 ± 4.69	93.64 ± 7.14	94.71 ± 5.92	94.84 ± 4.32	94.89 ± 6.74	94.72 ± 3.54	92.30 ± 5.52	93.52 ± 5.69
95.51 ± 3.86	94.89 ± 5.46	95.96 ± 4.56	94.18 ± 6.67	94.39 ± 4.86	95.94 ± 2.76	96.02 ± 5.78	95.84 ± 3.62	94.17 ± 4.80	94.93 ± 3.51

**Table 3:** The table shows the mean and the standard deviation of the sip volumes ingested by each subject. The last column shows the estimation error when using the mean as the predicted swallowed volume.

Subject	Mean (mL)	SD (mL)	Error (%)
F20	23.83	5.13	15.35
F22	19.42	5.17	22.94
F28	8.73	3.05	29.05
M20	12.19	4.33	34.10
M21	11.40	3.71	30.04
M211	13.67	3.15	17.34
M25	18.72	6.14	28.50
M251	7.14	2.96	40.73
M27	12.73	3.98	28.01
M29	15.13	5.99	32.71
M67	21.33	8.75	43.67
Across All	14.93	5.29	29.31

**Table 4:** The table shows how the RMSE and the average estimation error change for LR as features are added. As performance did not improve with the addition of the second feature, it was not deemed necessary to proceed with the addition of further features.

Features	RMSE (mL)	Average Estimation Error (%)
ASM	3.90 ± 1.58	24.63 ± 7.03
ASM, TM4	3.98 ± 1.60	25.11 ± 8.07

**Table 5:** The table shows how the RMSE and the average estimation error change for the ANN as features are added. Performance deteriorated with the addition of a third feature; thus, it was not deemed necessary to proceed with further feature addition.

Features	RMSE (mL)	Average Estimation Error (%)
SSC	3.84 ± 2.52	19.35 ± 11.60
SSC, MPD	2.80 ± 1.22	15.43 ± 8.64
SSC, MPD, VAR	3.45 ± 1.71	16.80 ± 6.76

## Data Availability

Raw data are available for sharing if requested.
